# *Nocardia* polymerase chain reaction (PCR)-based assay performed on bronchoalveolar lavage fluid after lung transplantation: A prospective pilot study

**DOI:** 10.1371/journal.pone.0211989

**Published:** 2019-02-25

**Authors:** Julien Coussement, David Lebeaux, Najla El Bizri, Vincent Claes, Michel Kohnen, Deborah Steensels, Isabelle Étienne, Hélène Salord, Emmanuelle Bergeron, Veronica Rodriguez-Nava

**Affiliations:** 1 Department of Infectious Diseases, CUB-Hôpital Erasme, Université Libre de Bruxelles, Brussels, Belgium; 2 Department of Microbiology, CUB-Hôpital Erasme, Université Libre de Bruxelles, Brussels, Belgium; 3 Service de Microbiologie, Unité Mobile de Microbiologie Clinique, Assistance Publique-Hôpitaux de Paris, Hôpital Européen Georges Pompidou, Université Paris Descartes, Sorbonne Paris Cité, Paris, France; 4 Lung Transplantation Unit, CUB-Hôpital Erasme, Université Libre de Bruxelles, Brussels, Belgium; 5 Laboratoire de Bactériologie, Hôpital de la Croix-Rousse, Lyon, France; 6 Research Group on Bacterial Opportunistic Pathogens and Environment, UMR CNRS5557, INRA1418 Écologie Microbienne, Observatoire Français des Nocardioses, Hospices Civils de Lyon, France, Université de Lyon 1, VetAgro Sup, Lyon, France; University of Toledo, UNITED STATES

## Abstract

**Background:**

Transplant recipients are at risk of pulmonary nocardiosis, a life-threatening opportunistic infection caused by *Nocardia* species. Given the limitations of conventional diagnostic techniques (i.e., microscopy and culture), a polymerase chain reaction (PCR)-based assay was developed to detect *Nocardia* spp. on clinical samples. While this test is increasingly being used by transplant physicians, its performance characteristics are not well documented. We evaluated the performance characteristics of this test on bronchoalveolar lavage (BAL) fluid samples from lung transplant recipients (LTRs).

**Methods:**

We prospectively included all BAL samples from LTRs undergoing bronchoscopy at our institution between December 2016 and June 2017 (either surveillance or clinically-indicated bronchoscopies). Presence of microbial pathogens was assessed using techniques available locally (including microscopy and 10-day culture for *Nocardia*). BAL samples were also sent to the French Nocardiosis Observatory (Lyon, France) for the *Nocardia* PCR-based assay. Transplant physicians and patients were blinded to the *Nocardia* PCR results.

**Results:**

We included 29 BAL samples from 21 patients (18 surveillance and 11 clinically-indicated bronchoscopies). Nocardiosis was not diagnosed in any of these patients by conventional techniques. However, *Nocardia* PCR was positive in five BAL samples from five of the patients (24%, 95% confidence interval: 11–45%); four were asymptomatic and undergoing surveillance bronchoscopy, and one was symptomatic and was later diagnosed with influenza virus infection. None of the five PCR-positive patients died or were diagnosed with nocardiosis during the median follow-up of 21 months after the index bronchoscopy (range: 20–23 months).

**Conclusions:**

In this prospective study, *Nocardia* PCR was positive on BAL fluid from one fourth of the LTRs. *Nocardia* PCR-based assays should be used with caution on respiratory samples from LTRs because of the possible detection of airway colonization using this technique. Larger studies are required to determine the usefulness of the *Nocardia* PCR-based assay in transplant recipients.

## Introduction

Nocardiosis is an invasive infection caused by *Nocardia* species (spp.), which are Gram-positive branching filamentous bacteria [1]. As most *Nocardia* infections arise by inhalation of this environmental bacterial organism, the lung is the organ most commonly involved in patients with nocardiosis [2, 3].

Nocardiosis generally affects compromised hosts, such as transplant recipients, cancer patients or those with autoimmune diseases [4]. Lung transplant recipients (LTRs) carry one of the highest risks of nocardiosis, with an estimated incidence of 0.8–3.5% (as compared with < 0.1% in the general population) [4, 5]. Several elements may explain this finding, including the use of immunosuppressive drugs (to prevent allograft rejection) in these patients and impaired bronchial mucociliary clearance and cough reflex (as a consequence of graft denervation). Also important is the direct exposure of the graft to environmental organisms such as *Nocardia* spp.

Diagnosing pulmonary nocardiosis is challenging [1, 6]. Clinically, *Nocardia* pulmonary infections resemble the pulmonary infections seen with many other infectious pathogens including bacterial and fungal organisms [1, 2]. Microbiologically, the two conventional diagnostic techniques (i.e., microscopy and culture) have a number of limitations. First, suggestive branching filamentous organisms may not be visible under microscopy despite the presence of culture-proven nocardiosis [1, 6]. Second, cultures may be negative if the incubation time is not long enough (e.g., < 2 weeks, although most isolates grow within one week), when appropriate media are not used, and/or when active antimicrobial therapy has already been started before sampling [1, 4].

To overcome these drawbacks, a polymerase chain reaction (PCR)-based assay was developed for the detection of *Nocardia* spp. on clinical samples [7]. Similar to other molecular tools that are routinely used in transplant recipients with suspicion of pulmonary infection (e.g., PCRs targeting *Pneumocystis jirovecii*, *Mycobacterium tuberculosis*, or community-acquired respiratory viruses), a *Nocardia* PCR-based assay may help transplant physicians to identify *Nocardia* in a rapid and culture-independent manner. In the recent years, the *Nocardia* PCR-based assay has been increasingly used in routine clinical practice in France and, currently, around 400 tests are performed each year by the French Nocardiosis Observatory. However, a prerequisite for larger routine use of the *Nocardia* PCR-based assay in the transplant population is determination of the performance characteristics of the test. Although initial findings suggested good specificity with negative results obtained in all 20 samples collected from patients without nocardiosis [7], some of us participated in a study that more recently suggested that *Nocardia* PCR may be positive in immunocompromised hosts without *Nocardia* infection [8].

To assess the performance characteristics of the *Nocardia* PCR-based assay for the diagnosis of pulmonary nocardiosis in LTRs, we evaluated all bronchoalveolar lavage (BAL) fluid samples prospectively collected from LTRs undergoing bronchoscopy in our institution.

## Material & methods

### Study design and ethics

We conducted a single center, prospective, pilot study at Erasme Hospital (Brussels, Belgium) between December 2016 and June 2017. All LTRs undergoing a bronchoscopy with BAL during the study period were included. Bronchoscopies were either surveillance procedures (i.e., bronchoscopies performed at pre-determined time-points during the usual follow-up after transplant) or clinically-indicated (e.g., for suspicion of pulmonary infection or graft rejection). All BAL fluid samples were initially analyzed locally to assess the presence of microbial pathogens (using methods available at Erasme Hospital, as described below, including microscopy and culture for *Nocardia*). Samples were also sent to the French Nocardiosis Observatory (Lyon, France) for *Nocardia* PCR testing. Transplant physicians and patients were blinded to the *Nocardia* PCR results. Our research was approved by the ethical committee of the Erasme Hospital (Erasme-ULB, ref: P2015/023). Need for consent was waived by the ethics committee given the nature of the study.

### Study setting

Erasme Hospital is an academic center at which 15–20 lung transplants are performed annually. At the time our study was conducted, approximately 200 LTRs were under active follow-up. In the last 15 years, a mean number of one case of post-organ transplant nocardiosis was managed each year at our institution [2].

### Conventional techniques used locally to assess the presence of *Nocardia* (i.e., microscopy and culture) and other respiratory pathogens

After receipt, BAL fluid samples were immediately analyzed at Erasme Hospital to assess the presence of *Nocardia* using conventional techniques. First, samples were assessed using microscopy with Gram staining to detect the presence of Gram-positive branching filamentous organisms evocative of *Nocardia spp*. Second, samples were cultured using Buffered Charcoal Yeast Extract–Glycin Vancomycin Polymyxin Cycloheximide (BCYE-GVPC) agar plates (Bio-Rad) and 10-days incubation (temperature: 35–37°C).

BAL fluid samples were also analyzed locally to assess the presence of pathogens other than *Nocardia*. For all samples (from surveillance and clinically-indicated bronchoscopies), the local laboratory routinely assessed the presence of common bacterial pathogens (using microscopy with Gram stain, as well as 2-day cultures on Columbia agar supplemented with 5% sheep blood [Becton Dickinson] and *Haemophilus* Chocolate 2 agar [BioMérieux]), mycobacteria (using microscopy with auramine stain, and 56-day culture using the BACTEC Mycobacteria Growth Indicator Tube [MGIT] 960 system [Becton Dickinson]), community-acquired respiratory viruses (using rapid antigen detection tests for influenza and respiratory syncytial virus [RSV], and viral culture), and fungal pathogens (using microscopy with Giemsa and Calcofluor stains, as well as 2-week culture on Sabouraud agar with gentamicin and chloramphenicol + 5% sheep blood [Becton Dickinson]). Additional tests were performed upon request of the transplant physician in patients in whom specific pulmonary infections were suspected (e.g., galactomannan antigen and simplex PCRs targeting *Pneumocystis jirovecii*, *Legionella pneumophila*, *Mycobacterium tuberculosis*, *Toxoplasma gondii*, cytomegalovirus [CMV], herpes simplex virus [HSV], Epstein-Barr virus). Some BAL samples were also tested using a customized Taqman Array Card real-time PCR method simultaneously targeting several pathogens (influenza virus, human metapneumovirus, parainfluenza viruses, rhinovirus, adenovirus, coronaviruses, bocavirus, enterovirus, CMV, HSV, RSV, human herpesvirus 6, *Bordetella pertussis*, *Chlamydia psittaci*, *Coxiella burnetii*, *Mycoplasma pneumonia*, *Legionella pneumophila* and *Pneumocystis jirovecii*) [9].

### *Nocardia* PCR and sequencing

After bronchoscopy, 1 mL of each BAL fluid sample was stored at -20°C for further characterization. These aliquots were subsequently sent to the French Nocardiosis Observatory to assess the presence of *Nocardia* DNA. DNA was extracted using the MTB respiratory specimen preparation Kit (Roche).

The *Nocardia* 16S ribosomal ribonucleic acid (16S rRNA) PCR-based assay was performed on all BAL samples. For this purpose, NG1 (5’-ACCGACCACAAGGGG-3’) and NG2 (5’-GGTTGTAACCTCTTCGA-3’) primers were used to detect a *Nocardia* genus-specific 590-bp fragment of the 16S rRNA gene. We used non-diluted and, to reduce the concentration of inhibitors, diluted DNA samples [8]. Other characteristics of this non-quantitative PCR-based assay, which was developed by our group, have been described elsewhere [8].

If the *Nocardia* PCR was positive, a second PCR targeting the *hsp65* gene was performed to provide species identification. The *hsp65* gene was selected because the 16S rRNA gene fragment amplified in the PCR described above is not considered polymorphic enough to allow accurate identification at the species level. For this purpose, TB11 (5’-ACCAACGATGGTGTGTCCAT-3’) and TB12 (5’-CTTGTCGAACCGCATACCCT-3’) primers were used to detect a 441-bp fragment of the *hsp65* gene encoding the 65-kDa heat shock protein [10]. PCR program and reaction mixture were carried out as described elsewhere [11]. The positive PCR products were sequenced using Sanger technology (Biofidal). Sequences were compared with those stored in GenBank using blast alignment software (http://www.ncbi.nlm.nih.gov/BLAST) and BIBI (Bio Informatic Bacteria Identification tool; http://umr5558-sud-str1.univ-lyon1.fr/lebibi/lebibi.cgi). Identification at the species level required 99% sequence similarity with the type strain of a single species.

### Clinical data collected

We collected demographic information (sex, age), transplant-related parameters (type of lung transplant, indication for transplantation, type of donation, CMV serostatus, occurrence of biopsy-proven acute rejection in the last six months), BAL-related characteristics (surveillance vs. clinically-indicated bronchoscopy, time from transplant to bronchoscopy, BAL fluid leukocyte count, microscopy and culture results, other microbiological findings), treatment details at time of bronchoscopy (immunosuppressive regimen, use of antibiotics), blood test findings (white blood cell count, C reactive protein concentration, kidney function, immunoglobulin G concentration), and outcome data (occurrence of nocardiosis, allograft rejection or death following bronchoscopy). Low-dose cotrimoxazole was defined as no more than 800/160 mg daily.

### Statistical analysis

The final analysis was performed after all data were verified. Categorical variables are presented as numbers and frequencies. Continuous variables are presented as medians (extreme values). *Nocardia* PCR sensitivity was calculated as the proportion of patients with pulmonary nocardiosis (see definition below) who had a positive PCR result. Specificity was calculated as the proportion of patients without pulmonary nocardiosis who had a negative PCR result. Diagnosis of pulmonary nocardiosis was based on the association of (1) presence of signs and/or symptoms compatible with nocardiosis and (2) growth of *Nocardia* spp. from a respiratory sample.

## Results

### Study population

**Fig 1:**
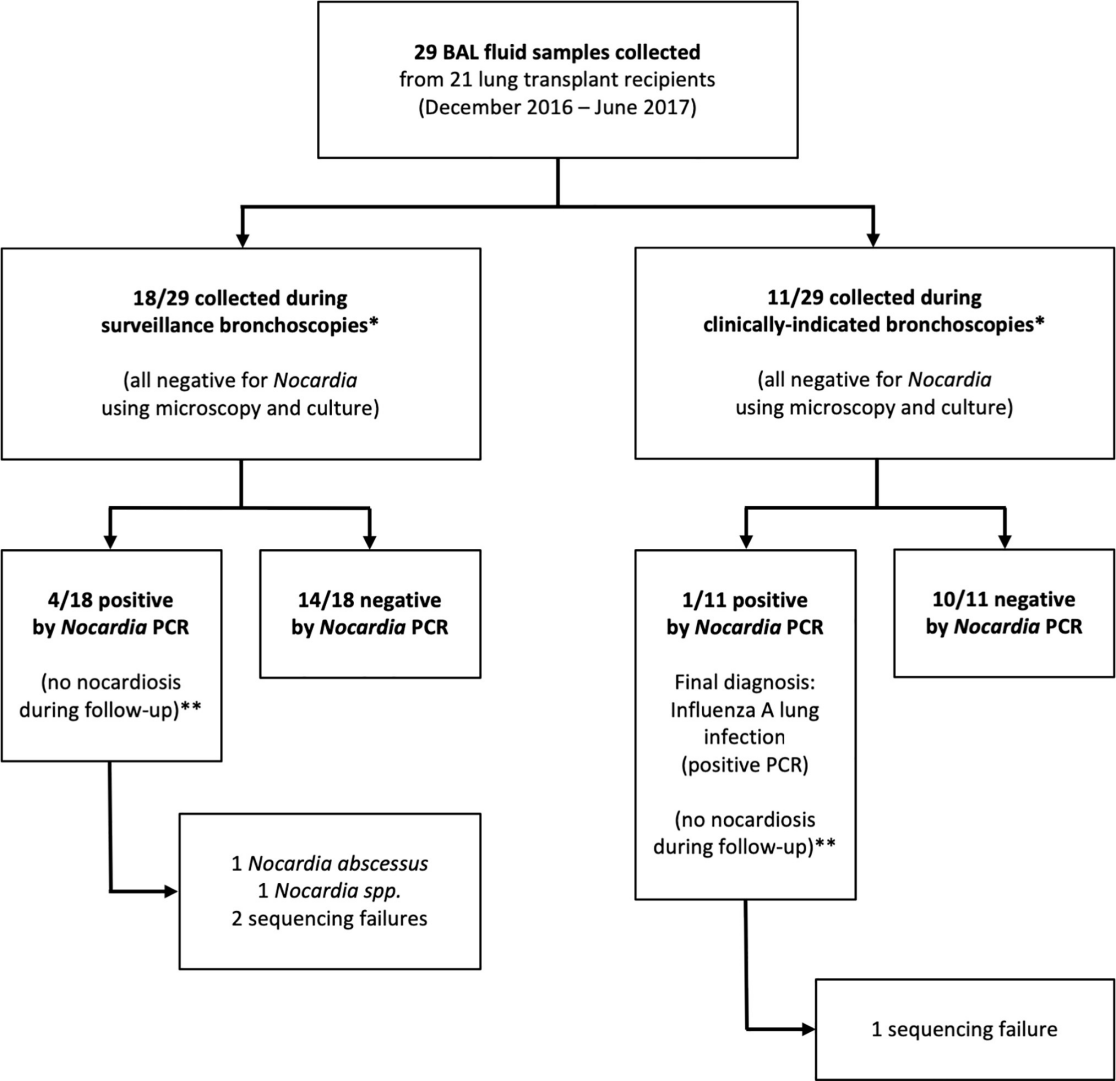
Flow chart. BAL: bronchoalveolar lavage; PCR: polymerase chain reaction. *Surveillance bronchoscopies were defined as procedures performed at pre-determined time-points during the usual follow-up of patients after lung transplantation; clinically-indicated bronchoscopies were defined as procedures done when there was a suspicion of graft rejection or lung infection. **The five PCR-positive patients were followed up for a median duration of 21 months after the index bronchoscopy (range: 20 to 23 months).

We collected 29 bronchoalveolar lavage (BAL) fluid samples from 21 lung transplant recipients (LTRs) who underwent bronchoscopy during the study period (**Fig 1**). This included 18 surveillance bronchoscopies (62%) and 11 clinically-indicated bronchoscopies (38%). Most patients had a single BAL fluid sample collected during the study period (15/21, 71%), but four patients had two BAL samples collected and two had three samples. One patient was a cardiopulmonary transplant recipient; the others were bilateral LTRs (95%, 20/21). Low-dose cotrimoxazole prophylaxis was ongoing in 23/29 cases (79%, weekly dose of sulfamethoxazole ≤ 1600 mg in all cases). Other characteristics of the included patients are shown in **Table 1**.

**Table 1:** Baseline characteristics of study patients.

	Total cohort (29 BALs in 21 patients)	PCR- BALs (24 samples from 18 patients)	PCR+ BALs (5 samples from 5 patients)
**Demographics**			
Male, n (%)	12 (57)	10 (56)	4 (80)
Median age at time of BAL (range)	56 (28–70)	57 (32–70)	56 (28–66)
**Transplant-related characteristics**			
Indication for transplantation			
COPD, n (%)	11 (52)	10 (56)	2 (40)
Cystic fibrosis, n (%)	4 (19)	2 (11)	3 (60)
Interstitial lung disease, n (%)	2 (10)	2 (11)	0
Other diseases, n (%)	4 (19)	4 (22)	0
Donation after brain death (vs. cardiac death), n (%)	15 (71)	12 (67)	5 (100)
CMV D+/R- serostatus, n (%)	2 (10)	2 (11)	0
Biopsy-proven acute rejection in last six months, n (%)	6 (21)	5 (21)	1 (20)
**BAL-related characteristics**			
Surveillance BAL (vs. clinically-indicated), n (%)	18 (62)	14/24 (58)	4/5 (80)
Median time from transplant to BAL (days) (range)	327 (10–5105)	299 (10–3892)	383 (187–5105)
Median BAL fluid leukocytes (/mm3) (range)	210 (30–7300)	220 (30–7300)	200 (80–650)
Median BAL fluid neutrophils (%) (range)	8 (0–91)	7 (0–91)	8 (0–64)
Median BAL fluid lymphocytes (%) (range)	4 (0–15)	3 (0–15)	5 (1–12)
Median BAL fluid macrophages (%) (range)	76 (5–97)	75 (5–97)	79 (29–82)
**Therapy at time of BAL**			
Tacrolimus (vs. cyclosporine A), n (%)	28 (97)	23 (96)	5 (100)
Anti-proliferative drug*, n (%)	11 (38)	7 (21)	4 (80)
Median daily dose of methylprednisolone (mg) (range)	8 (4–48)	8 (4–48)	8 (4–16)
Cotrimoxazole prophylaxis, n (%)**	23 (79)	19 (79)	4 (80)
**Blood tests at time of BAL**			
Median white blood cell count (x1000/μL) (range)	5.9 (3.2–20.8)	5.7 (3.3–20.8)	6.7 (3.2–7.1)
Median neutrophil count (x1000/μL) (range)	4.2 (2–14.5)	3.9 (2.1–14.5)	4.3 (2–5.1)
Median lymphocyte count (x1000/μL) (range)	1 (0.1–6.5)	1 (0.1–6.5)	1.1 (0.6–1.7)
Median CRP concentration (mg/L) (range)	3.8 (0–320)	4.8 (0–320)	1.1 (0–8.5)
Median eGFR (ml/min/1.73m2) (range)	63 (18–112)	62 (18–112)	68 (42–110)
Median immunoglobulin G concentration (g/L) (range)	7.1 (4.1–11.5)	7 (4.1–11.5)	7.5 (7–8.4)

**NOTE:** BAL: bronchoalveolar lavage; CMV: cytomegalovirus; COPD: chronic obstructive pulmonary disease; CRP: C reactive protein; D: donor; eGFR: estimated glomerular filtration rate (using CKD-EPI formula); R: recipient

*either mycophenolic acid or azathioprine

**weekly dose of sulfamethoxazole ≤ 1600 mg in all cases

### Conventional techniques (i.e., microscopy and culture) to detect *Nocardia spp*

Microscopy and culture showed no *Nocardia* spp. in any of the 29 samples (**Fig 1**).

### *Nocardia* PCR and gene sequencing

The 16S rRNA *Nocardia* PCR-based assay was positive in 5/29 BAL fluid samples from 5 different patients (24%, 95% confidence interval: 11% to 45%, using the Wilson method) (**Fig 1**). The *Nocardia* PCR specificity for diagnosis of pulmonary nocardiosis was therefore 83% (24 negative tests/29 samples from patients without pulmonary nocardiosis). Given that none of the patients had nocardiosis during the study period (based on culture), we were unable to determine the sensitivity of the PCR-based test. Among the 5/29 samples that were positive using the 16S rRNA PCR-based assay, the corresponding confirmatory *hsp65* PCRs yielded the following results: *N*. *abscessus* was identified in 1 sample (99.1% of similarity), *Nocardia* sp. was identified in another sample (85% similarity), and the 3 remaining reactions yielded negative results (PCR amplification failure).

### Characteristics of the five PCR-positive patients

The five positive samples were collected from four patients undergoing surveillance bronchoscopy and one patient undergoing clinically-indicated bronchoscopy (**Fig 1**).

Regarding the four patients undergoing surveillance bronchoscopy, none of them were receiving antibiotics active against *Nocardia* (other than low-dose cotrimoxazole) that would have masked the culture results regarding *Nocardia*. Transbronchial biopsy results were unremarkable in all four patients.

The single patient who had a clinically-indicated bronchoscopy presented with flu-like syndrome, cough and dyspnea. Amoxicillin-clavulanate was started and a bronchoscopy was performed. A diagnosis of influenza A H3 lung infection was made (influenza PCR-positive BAL fluid sample). Consequently, amoxicillin-clavulanate was stopped; anti-viral therapy with oseltamivir was initiated and symptoms resolved.

### Clinical follow-up of the five PCR-positive patients

The five PCR-positive patients were followed up for a median duration of 21 months after the index bronchoscopy (range: 20 to 23 months). None of these five patients died during the study follow-up. Using conventional techniques, pulmonary nocardiosis was not diagnosed in any of the PCR-negative patients during the follow-up, and there was no clinical event suggestive of nocardiosis. No allograft rejection was diagnosed during the study follow-up.

## Discussion

In this prospective study to evaluate the performance of the *Nocardia* PCR-based assay in LTRs, a fourth of our LTRs (5/21) had a positive *Nocardia* PCR on their BAL fluid samples without any element suggesting nocardiosis (microscopy, culture or six-month follow-up).

Although isolation of *Nocardia* spp. from a respiratory sample may theoretically indicate colonization [1], it is rare for *Nocardia* spp. to be identified in the airway of asymptomatic persons using conventional laboratory methods (i.e., microscopy and culture); indeed, in a retrospective study covering an 11-year period in a 1750-bed Spanish hospital, only six patients with *Nocardia* colonization were identified and all had severe underlying respiratory conditions [12]. Respiratory colonization with *Nocardia* has sometimes been described in patients with cystic fibrosis [13–16] or those who received a bone marrow transplantation [17]. In asymptomatic LTRs, it is very uncommon to culture *Nocardia* from a respiratory sample [1].

We identified five patients who had a positive *Nocardia* PCR on their BAL fluid samples without any element suggesting *Nocardia* infection. It seems very likely that these five positive tests represent *Nocardia* airway colonization. In fact, we are not the first to report molecular detection of *Nocardia* spp. in patients without evidence of nocardiosis. First, a study using PCR/electrospray ionization-time-of-flight-mass spectrometry showed that 46 of 101 healthy American soldiers had evidence of *Nocardia* colonization when screened with swabs from nares, oropharynx and groin [18]. Second, *Nocardia* PCR has been found to be positive in some patients with pulmonary tuberculosis and human immunodeficiency virus co-infection [19]. Third, a recent prospective study assessed the sensitivity (88%) and specificity (74%) of *Nocardia* PCR in at-risk patients with signs and symptoms compatible with nocardiosis. Among the negative controls (i.e., patients without nocardiosis, including 12 organ transplant recipients), *Nocardia* PCR was positive in 12/47 cases (26%) [8]. Interestingly, all these 12 positive PCRs were performed on respiratory samples from patients who most frequently had an underlying lung disease. Given that *Nocardia* is ubiquitous in the environment, we suggest that *Nocardia* spp. might be detected at the molecular level in the airways of LTRs. A parallel could be made with other environmental microorganisms, such as *Pneumocystis jirovecii* and *Aspergillus* spp., which often colonize (or infect) the airways of organ transplant recipients [20, 21].

The fact that some transplant recipients may have a positive *Nocardia* PCR on respiratory specimens in the absence of invasive nocardiosis indicates that, if this molecular tool is implemented, there is a risk of over-diagnosis of nocardiosis and consequent misuse of antibiotics. Importantly, the treatment of nocardiosis often requires the use of potentially toxic antimicrobial agents (e.g., high-dose cotrimoxazole, linezolid or aminoglycosides) and prolonged therapy (≥ six months in most cases). Moreover, antibiotic use is known to disrupt gut microbiota, select antimicrobial resistance and promote *Clostridium difficile-*associated diarrhea. For all these reasons, the *Nocardia*-based PCR assay should be used with great caution in respiratory samples from LTRs.

Although the management of LTRs who have a respiratory sample that is *Nocardia* PCR+/culture- still needs to be determined, it is reassuring that none of the five patients we identified in this situation died or received a diagnosis of nocardiosis or allograft rejection during the study follow-up (median duration of 21 months after the index bronchoscopy, range: 20 to 23 months).

There are several limitations to our study. First, this pilot study does not allow us to estimate the sensitivity of the *Nocardia* PCR-based assay given the lack of cases of nocardiosis during the study period. Given the relatively low incidence of nocardiosis after lung transplantation (estimated incidence 0.8–3.5%), similar studies involving multiple transplant units and larger numbers of transplant recipients are desirable. Second, it is possible that our incubation time of 10 days was not long enough to detect some *Nocardia* isolates. It has been suggested that cultures should be incubated for two weeks when looking for *Nocardia* spp. [1]; however, most isolates grow within one week [6]. Third, our *hsp65* PCRs did not allow species identification in 4/5 PCR-positive cases. It is relatively unclear why 3/5 patients had a negative *hsp65* PCR; it is possible that the primers used for the *hsp65* PCR were not sensitive enough to detect small quantities of nocardial DNA. Regarding the 1/5 patient who had a positive *hsp65* PCR not allowing species identification, it is possible that the strain belonged to an uncommon species for which *hsp65* do not allow adequate identification at species level.

In conclusion, one fourth of LTRs included in our study had a positive *Nocardia* BAL fluid PCR. The non-occurrence of nocardiosis during the study follow-up suggests that these results represented *Nocardia* airway colonization. We recommend that results of this *Nocardia* PCR-based assay in respiratory samples should be interpreted with great caution when used for diagnosis, given its lack of specificity for the diagnosis of invasive pulmonary nocardiosis. Larger studies are required to determine the usefulness of the *Nocardia* PCR-based assay in transplant recipients.

## Supporting information

S1 Table Dataset. (XLSX)

## Abbreviations

16S rRNA: 16S ribosomal ribonucleic acid
BAL: bronchoalveolar lavage
CMV: cytomegalovirus
*hsp65*: *heat-shock protein 65*
HSV: herpes simplex virus
LTR: lung transplant recipient
PCR: polymerase chain reaction
RSV: respiratory syncytial virus
spp.: species
